# Difficulties and Needs of Adolescent Young Caregivers of Grandparents in Italy and Slovenia: A Concurrent Mixed-Methods Study

**DOI:** 10.3390/ijerph19052837

**Published:** 2022-02-28

**Authors:** Sara Santini, Barbara D’Amen, Marco Socci, Mirko Di Rosa, Elizabeth Hanson, Valentina Hlebec

**Affiliations:** 1 Centre for Socio-Economic Research on Aging, IRCCS INRCA-National Institute of Health and Science on Aging, Via Santa Margherita 5, 60124 Ancona, Italy; s.santini2@inrca.it (S.S.); b.damen@inrca.it (B.D.); 2 Laboratory of Geriatric Pharmacoepidemiology, IRCCS INRCA-National Institute of Health and Science on Aging, Via Santa Margherita 5, 60124 Ancona, Italy; m.dirosa@inrca.it; 3 Department Health and Caring Sciences, Linnaeus University, SE-39182 Kalmar, Sweden; elizabeth.hanson@lnu.se; 4 The Swedish Family Care Competence Centre, Strömgatan 13, SE-39232 Kalmar, Sweden; 5 Faculty of Social Sciences, University of Ljubljana, Kardeljeva Ploščad 5, 1000 Ljubljana, Slovenia; valentina.hlebec@fdv.uni-lj.si

**Keywords:** adolescent young carers, caring difficulties, Italy, older people, long-term care systems, Slovenia, support needs

## Abstract

Many adolescent young caregivers (AYCs) care for a grandparent (GrP) with chronic disease, especially in countries with no or low developed long-term care systems and/or level of awareness of and policy responses to young caregivers. This mixed-methods study aimed at shedding light on the needs and difficulties faced by a sample of 162 adolescents aged 15–17, caring for GrPs, living in Italy (87) and Slovenia (75), respectively. A multiple linear regression model was built for the quantitative data. Qualitative data were content analysed using an open coding process. Italian and Slovenian respondents reported a moderate amount of caring activity and relatively high positive caregiving outcomes. Nevertheless, one out of three AYCs reported health problems due to their caring responsibilities. Compared to their Italian counterparts, Slovenian respondents were supported to a lesser extent by public services. Italian respondents faced communicative and practical problems; Slovenian AYCs experienced mainly emotional discomfort. AYCs from both countries requested emotional and practical support from formal services and family networks. Further, Slovenian AYCs requested emotional support and a personalized learning plan from schoolteachers. Support measures aimed at training AYCs of GrPs on geriatric care are recommended to address specific issues related to ageing and long-term care needs.

## 1. Introduction

### 1.1. Adolescent Young Caregivers of Grandparents

Adolescent young caregivers (AYCs) are youngsters aged 15–17 years who provide care to one or more family member(s) or close friend(s) and often carry out significant or substantial caring activities [1,2,3]. There are few cross-country and large sample research studies on (A)YCs. The figures reported in available national studies suggest that the prevalence of (A)YCs is between 2% and 8% of all children, young people, and young adults in advanced industrialized capitalist societies. Nevertheless, these are rough estimates of the prevalence of (A)YCs in the whole young population, because every national study adopted a different definition of caring and (A)YC, and a different methodology to identify and count them (e.g., the age range) [4]. However, results collected at the national level seem to suggest that caring for a sick or disabled family member during adolescence and youth is quite a common phenomenon [5]. Focusing on the two countries that are analysed in this study, while no estimate of the number of AYCs in Slovenia is available so far, in Italy 7% of the population between the ages of 15 and 24 are estimated to take care of frail adults or older people, i.e., grandparents (GrPs) [6]. 

AYCs of GrPs are under-represented in international social research [7]. 

The limited available literature highlights that they perform instrumental activities of daily living and provide companionship and emotional support [8,9]. Further, especially in multigenerational families, they can adopt the role of primary or auxiliary carers, depending on the presence of adults in the household who can play the role of primary carers [9].

AYCs (of GrPs) generally experience both positive and negative caregiving outcomes. Positive outcomes include soft skills such as maturity [10], resilience in the face of difficulties [11], empathy [12], and a better self-efficacy [13]. Conversely, negative outcomes include frustration and stress, mental health problems [14], and poor-well-being [15,16], in addition to the risk of experiencing inequalities in accessing health services [4,5,6,7,8,9,10,11,12,13,14,15,16,17], and of having fewer education and employment opportunities in the long-term [18,19,20]. AYCs often face numerous challenges at school, such as tardiness and/or absenteeism, and difficulties in completing or carrying out homework and meeting deadlines [21].

AYCs of GrPs seem to experience more positive caring outcomes in comparison to AYCs of other care recipients e.g., parents and siblings [9]. This could be related to the auxiliary role in providing support to parents who are primary caregivers, and due to feelings of affection towards grandparents and the desire to return the help received in childhood, which may mitigate the negative effects of caregiving [22].

### 1.2. Long-Term Care Systems and Policy Recognizing Adolescent Young Caregivers in Italy and in Slovenia

The involvement of minors in caring for an older family member is related to population ageing trends [23] and the connected increasing demand for long-term care (LTC) [24], especially in countries with less well-developed LTC systems, such as Italy and Slovenia. Italy has an LTC extensively based on a cash-for-care setup instead of in-kind services provision [25]. Slovenian LTC is characterized by a fragmented care provision based primarily on family members caring for older people living in the community and residential care managed by formal health and social care providers. [26]. Moreover, LTC systems in both countries are unable to ensure daily home care services to address the needs of frail older people [27,28,29].

In addition, the COVID-19 pandemic and the consequent interruption or decrease of many public and private services addressed to older people with LTC needs, e.g., home care and day-care centres [30,31], increased the care burden of their informal caregivers [32]. Among the latter, it is possible that adults’ difficulties in reconciling work and care responsibilities and the closure of schools confining adolescents at home have increased the involvement of young people in the assistance of older family members living in the same household [33]. This is in light of the evidence that cohabiting with an older person increases the likelihood of being involved in caring activities [34,35].

The awareness level of the role played by AYCs in society, the knowledge about the consequences of caring on AYCs’ lives, and the policies supporting AYCs tend to differ across Europe, as depicted by Leu and Becker [36]. According to their classification of countries, Italy is in the cluster of “emerging” countries, indicating a dearth of services for young carers, a lack of research, and low level of legal rights, recognition, and advocacy. Slovenia is classified as “awakening” due to a lack of research and embryonal awareness on informal carers, especially young carers. Moreover, in Italy and in Slovenia as well as in many European countries, the majority of health and care systems, when addressing young carers, still tend to work in silos, with a lack of integration of services and of cooperation between providers [37].

Neither Italy nor Slovenia has national legislation recognizing informal carers, regardless of their age; thus, the recognition and protection of AYCs is reliant upon “non-specific legislation” covering other more universal rights such as education, health and social care, and child safeguarding and protection legislation [38].

In Italy, a draft national law for the recognition and support of family caregivers presented in 2019 is still pending in the Senate [39] and the 2021 Financial Law established a Family Caregiver Fund, with an annual budget of 30 million for the three-year period of 2021–2023 for “legislative interventions for the recognition of the social and economic value of the non-professional care activity of the family care provider”. In the absence of national legislation, some Italian regions, e.g., Abruzzo, Campania, and Emilia-Romagna, have enacted laws that apply in their territories for sustaining family carers, providing support services that were extended in recent years to also include AYCs (in particular psychological or socio-educational interventions), as well as providing training interventions targeting health and social care professionals.

In Italy and Slovenia, support and protection to AYCs can be provided indirectly by legislation for the protection of people with disabilities and LTC needs and/or of a youngster’s childhood. In Italy, this is possible by providing support to care recipients, e.g., disabled older people. As monetary transfers prevail on in-kind services [40], the State Care Allowance established by the Law 18/1980 [41] guarantees approximately 500 €/month to people with disabilities. This amount can be spent on a discretionary basis by the beneficiaries, e.g., for purchasing support services, such as private home care services, and often for hiring live-in migrant care workers [42].

Moreover, AYCs’ health and well-being could be defended by the Law 451/1997 [43], establishing the Parliamentary Committee on Children and the National Observatory for Children, aimed to control the implementation of international conventions and legislation on the rights of the child, and by the Law 285/1997 [44] “Provisions for the promotion of rights and opportunities for childhood and adolescence”. Both laws are targeted towards children and adolescents without any age specifications.

In Slovenia, indirectly supporting legislation entails several laws. Among the latter there is the Social Security Act, which is the basis of services within the family [45]. Moreover, the drafted LTC legislation (pending in Parliament) is aimed at the integration and coordination of different aspects of treatment and at the promotion of care intended as a mutual responsibility between education, social, and health care systems. Furthermore, the Domestic Violence Prevention Act [46] includes measures to protect children and the Family Code [47] foresees provisions about how to protect the child and assess if the child is endangered.

In both countries, several services targeted at AYCs arise from international projects financed by the European Commission. For example, the “EDY-CARE” project (2017–2020, https://edycare.eu/, accessed on 9 January 2022) aimed at empowering teachers and school staff (e.g., school nurses, psychologists, social workers, management) working in upper secondary education to recognize AYCs, maximize their learning opportunities and strengthen their social inclusion. Further, the “Me-We” project (2018–2021, https://me-we.eu/the-project/, accessed on 9 January 2022) implemented and evaluated a psychoeducational intervention for increasing the resilience of AYCs in six European countries, including Italy and Slovenia. Nevertheless, generally, in the latter countries, dedicated training targeting teachers, social workers, and health professionals to enable them to recognise and reach out to AYCs, and dedicated support interventions for AYCs, continue to be rare. Also, the majority of Italian and Slovenian regional public social and health care services tend to ignore AYCs.

The lack of national laws, systems of identification for AYCs in schools, and sufficient funding make the enactment of policy and the design and implementation of support services highly challenging in both Italy and Slovenia. Thus, AYCs in both countries run the risk of falling through the gaps in political and legal safety nets and between services targeted at adults and children.

### 1.3. Aims of the Study

This study compares the situation of AYCs of GrPs in Italy and Slovenia that emerged from an online survey, carried out in six European countries within the mentioned EU funded project “Me-We”. Italy and Slovenia represented the two countries with higher numbers of AYCs of GrPs among the study respondents, whose percentages within the national samples were 36.3% and 28.3%, respectively, in comparison with 10.1% in the Swedish, 9.4% in the English, 9.4% in the Dutch, and 6.5% in the Swiss samples [22]. This outcome sparked the researchers’ interest to better understand the characteristics of the intergenerational caring relationship between nonadjacent generations (i.e., adolescent grandchild–grandparent) in the two countries with a higher prevalence of AYCs of GrPs. Thus, the data collected in the two countries were further analysed to determine the caring situation of this group of AYCs and to better understand to what extent the classification as an “emerging” country or an “awakening” one, according to Leu and Becker [36], mirrors different difficulties experienced by AYCs of GrPs and calls for specific policies and interventions.

To this purpose, this study aims at answering two research questions: (1) What are the difficulties and support needs of AYCs of GrPs in Italy and in Slovenia, respectively? (2) Which policies and support interventions should be implemented in each country, considering the level of awareness of and the policy responses and service supports available for AYCs?

## 2. Materials and Methods

### 2.1. Participants’ Inclusion Criteria and Recruitment Strategy

Data for this analysis form part of a large online survey carried out in 2018 and reaching 6119 youngsters in Italy, Slovenia, Sweden, Switzerland, the Netherlands, and the United Kingdom within the Me-We project that was aimed at increasing the knowledge about the mental health and well-being of European adolescents with caring roles.

Since this was the first major international survey mapping AYCs in Europe, the sole criteria for being included in the survey was being aged between 15 and 17 years and being available to fill in the questionnaire. In Italy as well as in Slovenia, due to the low level of awareness and the lack of specific social measures and interventions targeted at AYCs, the latter were recruited in high schools, as this was considered the environment in which as many teenagers as possible could be readily reached. In both countries, the schools selected for the recruitment were of different types in order to guarantee the participation of students with varied and heterogeneous socio-economic backgrounds.

School managers were contacted by the researchers and a meeting was arranged for explaining the purpose of the survey and for agreeing on times and procedures for conducting the survey.

In Italy, 17 upper secondary schools (10 in the Marche region in the centre of Italy and 7 in Emilia-Romagna in north-east Italy) were identified for being involved in the survey. Only two of them (both in the Marche region) refused to cooperate in the initiative (e.g., due to lack of time/difficulties in reconciling the organization of the survey with the training courses schedules, the unavailability of teachers). The 15 schools that were engaged in the recruitment were six lyceums, six technical schools, and three vocational schools.

In Slovenia, AYCs were recruited in 10 upper secondary schools, i.e., four general high schools, three vocational schools for health professionals, and three school centres that comprise various school programs, including general high schools and vocational schools for health and educational professionals, located in different regions across the country. Among the schools contacted to be involved in the study, only one high school for health professionals in Ljubljana refused to participate.

### 2.2. Data Collection Procedure and Ethics

Each data collection activity in both countries was preceded by a meeting with students during which the researchers introduced the topic of AYCs and what being an AYC means. This was necessary due to the scarcity of knowledge and information about caregiving at a young age.

In the two countries, data were gathered mainly through paper and pencil administration in light of the large number of students (in some cases more than 200 for each plenary meeting) and the limited number of electronic devices available. When the latter were available in a sufficient number, the survey was carried out mainly on personal computers available in the school’s informatics classroom(s) using the 1 ka online platform to guarantee participants’ anonymity and privacy.

Formal ethics approval was obtained in the spring of 2018: in Italy from the Alma Mater Studiorum at the University of Bologna, responsible for the data collection in the Emilia-Romagna (in Northern Italy) and the Marche region (in Central Italy), and in Slovenia from the Ethics Committee on Research at the Faculty of Social Sciences (KERFDV in Slovenian).

All respondents were recruited on a voluntary basis in accordance with the Declaration of Helsinki (1964; 2013) [48] and this was specified in the information letter accompanying the questionnaire and on the first page of the questionnaire. Participants were asked to give their consent to the data treatment and informed consent was also secured from parents/legal guardians, in accordance with applicable national legislation and institutional guidance. The data were processed in full compliance with both Italian and Slovenian laws on data protection and the General Data Protection and Regulation (EU 2016/679; Regulation, G.D.P.R., 2016) [49] to guarantee the youngsters’ anonymity and privacy.

AYCs were screened through two anchoring questions in the questionnaire. Then, within the whole sample of AYCs, a sub-sample of adolescents caring for GrPs was identified through a further anchoring question asking about the type of relationship with the cared-for person.

### 2.3. Study Design

A mixed-methods (MM) study design [50] was adopted to the purpose of understanding the experiences of AYCs of GrPs in Italy and in Slovenia, by coupling qualitative (QUAL) with quantitative (QUANT) findings such that the QUAL data could provide country-contextual data for enriching the interpretation of the QUANT data in each respective country. This study employs triangulation design, more specifically validating the QUANT data model [50], as open-ended questions were embedded within a larger quantitative survey. QUANT and QUAL data were analysed separately and then integrated during the process of contrasting and comparing the results of each phase. The QUANT data quantified the impact of caring on AYCs’ lives, e.g., AYCs’ health condition, wellbeing, and school performance. The QUAL findings provided the country’s context with regards to care and information on the condition of AYCs of GrPs living in the two countries, thus expanding and supporting the quantitative analysis.

### 2.4. Quantitative Measures and Qualitative Variables

The questionnaire included questions on demographic data, caregiving activity, impact of caring on respondents’ health and education, supports received, and three psychometric scales for assessing the level of caring activities and the impact of care on AYCs.

The quantitative outcomes measures in the study included the Multidimensional Assessment of Caring Activities (MACA) [51]; the Positive and Negative Outcomes of Caring (PANOC) [51]; the KIDSCREEN-10 [52,53] for measuring health-related quality of life; formal and informal supports received; and hours per day spent providing care.

MACA-YC18 is an 18-item self-report questionnaire asking young people about the type and frequency of their caring activities (items are rated on a 3-point scale where “never” = 0, “some of the time” = 1, and “a lot of time” = 2). An overall score is calculated ranging from 0 to 36. A score = 0 indicates that the respondent is not a caregiver; a 1–9 score means a low amount of care activity; a 10–13 score means a moderate caring activity; and a score of 14 and over indicates a high and very high amount of care.

PANOC-YC20 is a 20 item self-report measure that obtains indexes of positive (e.g., new relational skills, resilience, maturity and empathy, named PANOC-Pos) and negative outcomes (e.g., anxiety, frustration, sense of inadequacy, named PANOC-Neg) of care provision each ranging from 0 to 20. Each item is rated on a 3-point scale: “never” = 0, “some of the time” = 1, and “a lot of the time” = 2.

KIDSCREEN-10 is a 10-item measure of the health-related quality of life standard that provides a single score of HRQoL ranging from 10 (bad health) to 50 (good health). This measure was treated as a continuous variable.

AYCs were also asked to report if they suffered from health problems in connection to the care activity provided and to specify if these problems were physical (e.g., back pain, headache, muscle tension), psychological (e.g., anxiety, depression), or other.

The variable “health problems related to care” was born from a question asking the respondents to indicate if they suffered from any physical and/or psychological disorder due to care, e.g., back pain, headache, muscle tension, and/or anxiety and depression.

The non-standardized questions on health and choice options were identified based on the previous research studies on (A)YCs [1,2,3,4,5,6,7,8,9,10,11,12,13,14,15,16,17,18,19,20,21,22].

The variable “formal support” includes statutory agency/governmental supportive programs, services, and state monetary benefits targeted at the AYC and/or the older care recipient, while the variable “informal support” refers to help from other family members, friends, and neighbours. The assessment of formal and informal support was triangulated with the open-ended items described below.

The qualitative data were derived from two open-ended questions: (1) What can support you in your role of caregiver? (2) If you are caring for an older person (aged 65 and over), which are the main difficulties you are facing?

### 2.5. Analysis Methodology

Concerning QUANT measures, continuous variables were reported as mean and standard deviation (SD); comparison of variables between groups were performed by unpaired Student’s *t*-test or one-way analysis of variance (ANOVA), as appropriate. Categorical variables were expressed as the absolute number and percentage and statistical significance was assessed by Pearson’s Chi-square test. The statistical significance for this study was set at *p* < 0.05. Statistical analyses were conducted using the Stata 15.1 Software Package for Windows (StataCorp., College Station, TX, USA).

The QUAL responses were content analysed by applying an open coding process [54], a method through which concepts and their dimensions are identified and discovered directly from the qualitative data. Following this process, categories referring to the same phenomenon were grouped into sub-categories and these were subsequently grouped into higher-order categories, with the support of MAXQDA 2020 software to provide a clear description of the findings with a focus on a comparison between the Italian and Slovenian AYC respondents.

The trustworthiness of the qualitative elements of the study (i.e., credibility, dependability, transferability) was reached by ensuring methodological rigor and internal process assessment as recommended by literature on social sciences research methodology [55,56,57]. First, credibility was obtained by formulating open-ended questions on the basis of the available literature on (A)YCs that allowed the respondents to give an overall description of their care situation, by using simple (non-technical) language such that it was fully understandable by young people, and through frequent debriefing sessions among researchers within national teams and peer scrutiny. The dependability was reached through the detailed description and plan of the study design, which foresaw the comparison of QUAL with QUANT data. The study transferability was obtained through the study of previous research on this topic and the results were meant as a basis for further research.

## 3. Results

### 3.1. Sample Description

The whole sample was comprised of 162 AYCs caring for GrPs: 87 from Italy and 75 from Slovenia, respectively. Female participants represented 79% of the whole sample with a statistically significant difference between the two study countries. In fact, female respondents constituted more than 93% of the sample in Slovenia compared to 66.7% in Italy. In Italy, the AYCs were mainly aged seventeen years (48.3%), while in Slovenia they were aged sixteen years (53.3%).

In both Italy and Slovenia, AYCs were born in the same country where they resided. A total of 64.0% of Slovenian respondents lived with their grandparent(s) compared to 19.5% of Italians.

Italian and Slovenian AYCs provided 2.7 and 2.3 h of care per day to their GrPs, respectively (Table 1).

The most common diseases among the GrPs of the surveyed AYC, were physical disability (more than 62% in both countries), cognitive impairment (35.6% in Italy and 25.3% in Slovenia), and mental illness (20.7% in Italy and 24% in Slovenia), without any statistical significance.

### 3.2. Quantitative Results

Approximately one-third of Slovenian and Italian AYCs reported physical and/or psychological health problems as a consequence of their caregiving activities (Table 2).

Italian and Slovenian AYCs of GrPs reported a medium caring activity (MACA) score ranging from 10 to 13, but the Italians were at the lower limit of the range, while the Slovenes slightly exceeded it so that the difference between the two groups is statistically significant, indicating that young Slovenians had a greater care burden than Italian AYCs.

Interestingly, positive caregiving outcomes (PANOC-Pos) prevailed over negatives ones in both the sample groups and Slovenian AYCs experienced more positive caregiving outcomes than Italian AYCs (*p* = 0.048).

The KIDSCREEN-10 score indicates that both Italian and Slovenian AYCs felt quite happy, physically healthy, and satisfied with regards to family life, peers, and school life, but Slovenian AYCs reported a lower score than Italian AYCs, even if with a slight statistical significance.

Caregiving activity influenced the AYCs’ school attendance and performance only marginally in the two countries. However, nearly twice the percentage of Slovene AYCs had problems in attending school as a result of caring responsibilities compared to the Italian AYCs.

It is noteworthy that more than 73% of Slovenian and nearly half of Italian respondents reported were not receiving any formal support. However, among AYCs of GrPs receiving formal support, those living in Slovenia did not receive as many formal supports compared to AYCs in Italy (the difference is statistically significant, *p* = 0.003). Only approximately one-third of the two sample groups received informal help, e.g., from other family members and neighbours (Table 2).

Further analysis of those receiving formal or informal support (Table 3) revealed that there were no significant differences in the health status among Italian AYCs receiving formal support as opposed to those not receiving formal support in Italy. Moreover, AYCs in Italy receiving formal support reported significantly less hours of care than those not receiving formal support.

On the contrary, in Slovenia, AYCs receiving formal help reported more health problems, provided more hours of care than AYCs who do not receive formal support, and had more negative caregiving outcomes (PANOC-Neg). Moreover, AYCs in Slovenia receiving formal support also received informal support significantly more often than AYCs who did not receive formal support.

The analysis also showed that Slovene AYCs receiving informal help reported more health problems (*p* = 0.032) and received less formal support than AYCs not receiving informal help (*p* = 0.000). Italian AYCs of GrPs receiving informal help reported a lower KIDSCREEN score, i.e., a worse quality of life, compared to AYCs not receiving informal help and the difference is statistically significant (*p* = 0.032).

### 3.3. Qualitative Results

As outlined above, the qualitative data were derived from two open-ended questions: one was focused on the help that would support the respondents as a carer, while the second was focused on the main difficulties encountered by AYCs in providing assistance to GrPs.

The analysis shows that the surveyed Italian AYCs mostly required material support (13 quotations) including general and physical help, financial support, and information and training, while Slovenian AYCs needed emotional and moral support (13 quotations), as reported in Table 4. Concerning the difficulties encountered, consistent with the support needs, Italian AYCs experienced material and communication difficulties, with 41 and 29 quotations reported, respectively, while Slovenian AYCs mainly underlined material and emotional difficulties, as stated by the 15 and 4 quotations, respectively, as reported in Table 5.

Furthermore, the analysis highlighted that all respondents faced material and emotional strains, regardless of the country of residence, while two subcategories were specific to Slovenian AYCs: “School problems”, related to the category “Material difficulties”, and “Difficulties in managing behavioural characteristics”, included in the category “Emotional and psychological difficulties” (Table 5).

#### 3.3.1. Support Needed by AYCs of GrPs

With regard to the support needed by AYCs of GrPs, the analysis highlighted four main categories: “Support not required or undefined”, “Material support”, “Emotional and moral support”, and “Other supports”.

The category named “Support not required or undefined”, as shown in Table 4, included two subcategories named, respectively, “Don’t know”, that expressed uncertainty about what the support could be, and “Support not required”. The latter included some respondents that perceived that they were not in need of support as a consequence of being an auxiliary or temporary caregiver, as depicted by the following quotation:

“*I’m a semi-temporary caregiver. I carry out assistive activities by supporting my grandparents who, due to their age, are unable or find difficult to carry out certain activities in their daily life. Currently, I’m mainly concerned with my grandfather, who is carrying out rehabilitative activities following a major fall. Therefore, I don’t think I need any further support.*”

(Italian respondent).

*“I don’t have a big carer role because my grandfather doesn’t have many care needs”*

(Slovenian respondent).

Other respondents, both Italians and Slovenian, stated that their willingness to take on a caregiving role was due to their desire to help or due to the affective relationship with their grandparent(s). As a result, they did not feel the need for any kind of support:

“*Nothing, I do it because I grew up with that person and I don’t want to abandon her despite it being a degenerative disease. Nothing is needed when things are done with the heart.*”

(Italian respondent).

“*It was never difficult for me to help someone who needs my help. I always try to the best of my abilities. If someone helps me with this it´s his decision and his good will*”

(Slovenian respondent).

The second category arising from the analysis is the need for “Material support” (Table 4), that included subcategories of “General support”, “Financial support”, and “Physical support” related to the Italian respondents only. The quotations included in the subcategory “General support” stated the need for generic help provided by an unspecified person and role:

*“Someone to help me assist the sick person”*

(Italian respondent).

The need for “Financial support” expressed the need of a recognition of the AYC’s role, while the “Physical support” is related to the need of practical support in carrying out daily activities, i.e., getting up and moving:

“*Financial support from the state since the merits and above all the great sacrifice of a boy who puts the lives of others first than his must be recognized*”

(Italian respondent).

“[A support to] *Help them do what they aren’t able to do, for example accompany them to go out, help them to sit and to get up*”

(Italian respondent).

As shown in Table 4, the two subcategories “Information advice” and “Experts’ advice” included quotations related to the need for information that could be more general or be provided by an expert, as highlighted in the following sentences:

“*I would like to receive advice on how to better organize my day’s activities*”

(Italian respondent).

“*I would like to be well informed about what I´m going to do. To be informed about the possible complications or problems that I can stumble upon*”

(Slovenian respondent).

*“In the role of caregiver I would need a doctor with me to know how to help my grandma with the disease”*

(Italian respondent).

*“That I would get some advice from a person that is experienced in this field”*

(Slovenian respondent).

The third major category identified was the need for “Emotional and moral support”, which was related particularly to the Slovenian respondents (Table 4). These respondents reported needing “Emotional support”, referring to support from a generic person who could be both within and outside the family network (i.e., the subcategory “External or interpersonal support”):

“*Moral support*”

(Italian respondent).

“*A little company and a form of empathy from the teachers would help me*”

(Italian respondent).

*“For me to feel more useful”*

(Slovenian respondent).

“*My support could be, or rather they are, my good friends who I can confide in when it gets hard for me*”

(Slovenian respondent).

The final category is named “Other support” (Table 4) and was specific to the Italian respondents. It included respondents reporting the need for support to improve awareness on AYCs and their activities (i.e., subcategory “Recognition/legislation support”), the need for “Educational support”, and the need for mental health support services.

#### 3.3.2. Difficulties Encountered by AYCs of GrPs

The most prevalent difficulties are related to “Material difficulties”, which were classified into seven subcategories, respectively (Table 5).

As shown in Table 5, only two subcategories were reported by both Italian and Slovenian respondents, in particular related to “Difficulties in helping the care recipient with moving and handling” and “Difficulties in managing therapy”. The latter included quotations that expressed specific complications that emerged in managing their care recipient’s therapy:

“*Changing the Stoma bag*”

(Slovenian respondent).

“*Insecurities about the right medicines to make my grandfather take*”

(Italian respondent).

“Difficulties in helping the care recipient with moving and handling”, related to the relationship between AYCs and GrPs, highlights the physical burden as a consequence of the age/weight gap and of the difficulty to move a disabled and “uncooperative” grandparent safely:

*“Her body weight is heavy for me and the smell of certain leakages is disgusting”*

(Italian respondent).

“*The main problem is that I have problems lifting a person, that’s why I also have back problems*”

(Slovenian respondent).

The sub-categories “lack of time”, “financial difficulties”, “lack of information”, and “difficulties in helping parent’s life-work balance” were specific to the Italian respondents. These often referred to practical challenges and logistical aspects, as shown in the following quotations:

*“I haven’t enough time”*

(Italian respondent).

“*I think the Government should help more informal caregivers because you can often feel that you’re not able to assist someone else due to financial constraints e.g., treatments, facilities, visits, medicines, etc. …)*”

(Italian respondent).

“*What I miss more is the lack of information*”

(Italian respondent).

“*Sometimes I also go to work with mom to help her and so it’s difficult to combine study, care and work*”

(Italian respondent).

Finally, Slovenian respondents reported difficulties in the management of school life, as expressed by the sub-category “School problems”:

“*When I want to study someone always bothers me and that’s when I lose my concentration*”

(Slovenian respondent).

The second category concerns communication difficulties, reported by 29 Italian AYCs (Table 5); it includes difficulties in “conversing and talking” together with the care recipient(s) and difficulties in “understanding” the care recipient’s requests and problems. These expressed difficulties are closely connected with the caregiving relationship, the age gap, and the care recipient’s illness (e.g., cognitive impairments or hearing difficulties) as depicted by the following quotations:

“*Repeating things over and over to make them understand*”

(Italian respondent).

“*Understanding their problems and understanding how to help them not to think about it*”

(Italian respondent).

“*I find it difficult to understand my grandma: she has speech and hearing difficulties*”

(Italian respondent).

The category “Emotional and psychological difficulties” included four different sub-categories, one reported by both Italian and Slovenian respondents, in particular “Difficulties in supporting the care recipient’s mood” (Table 5), clearly described by the following quotations:

“*Age, it’s worse by the years. Everything hurts her and together with her I feel sad for her pain too*”

(Slovenian respondent).

“*It’s difficult for me making my grandparents smile really*”

(Italian respondent).

The subcategories “Feeling of discomfort, sadness, and guilt” and “Fear of not managing to take care” were specific to the Italian respondents and are, respectively, concerned with emotions of sadness related to the care recipient’s illness and with the fear of not being able to provide good care:

“*I simply feel deeply sad to see my paternal grandparents in the state they are, suffering from dementia*”

(Italian respondent).

“*I’m worried that I can’t take good care of them*”

(Italian respondent).

The “Emotional and psychological difficulties” also included “Difficulties in managing behavioural characteristics”, which was specific to the Slovenian respondents:

“*Grandmother is sometimes grumpy if all things aren’t as she says, she complains a lot if I don’t have time to, and my brother could help because he’s on his computer most of the time, (she) is mad at me, insults me; she doesn’t think of the help from my younger brother*”

(Slovenian respondent).

Finally, the analysis highlighted the category named “unspecified difficulties”, reported solely by Slovenian respondents, which included quotations that expressed the presence of difficulties without specifying them:

*“It’s difficult for me limiting the risk that my grandma could do something wrong for herself and she might get hurt”*

(Slovenian respondent).

## 4. Discussion

This study hypothesized that the phenomena of AYCs caring for GrPs is prevalent especially in countries with less well developed LTC systems and with a lower level of awareness, supports, and policy targeting informal carers in general and especially adolescent and young people covering caring roles within the household. This is the case for Italy and Slovenia, the two countries of focus in this study, classified as “emergent” (Italy) and “awakening” (Slovenia) [36]. Since this study is based on data collected through a non-representative survey involving a convenience sample of AYCs, its results cannot be generalized, i.e., it cannot prove that the prevalence of AYCs of GrPs is always higher in every country with a lack of a well-developed LTC system and a low level of awareness. Nevertheless, this was not the aim of this study, which was rather to broaden the knowledge about the intergenerational caring relationship between nonadjacent generations (i.e., grandchild–grandparent) in the two study countries. However, the correlation between family morals, ethical obligations, and LTC support services provided, along with the probability that adolescents take on the role of carers of their GrPs, is still an under-developed topic that is worthy of further study.

The mixed-method (MM) approach has proved suitable for exploring the complex and little-known phenomenon of intergenerational caring between adolescent grandchildren and GrPs. On the one hand, in fact, quantitative analysis has provided a comparative picture of the situation of a sample of AYCs in the two countries. On the other hand, the qualitative analysis provided input on “how” a group of adolescents experienced caring for a grandfather/mother with LTC problems and the nature of their support needs.

To the best of our knowledge, this is one of the first MM studies giving voice to AYCs of GrPs, framing the outcomes in the LTC systems and interpreting findings in light of the low level of awareness and dearth of support measures and policies for this group of informal carers.

### 4.1. Difficulties and Support Needs of AYCs of GrPs in Italy and Slovenia

Several differences emerged from the MM analysis between the two national samples.

In the Slovenian sample, living with GrPs was more common than in the Italian one, and probably this situation increased the likelihood for Slovenian AYCs to be involved in caregiving activities of their GrPs [58]. Since Italian and Slovenian AYCs provided a small number of hours of care per day (2.5 h a day on average), and some of them defined themselves as “semi-temporary” carers, we argue that they could mainly play the role of auxiliary carers, especially Slovenian respondents, who are more likely to live in multigenerational households that may include older family members with Alzheimer’s disease or dementia [59,60].

Caregiving for a GrP in general had more positive than negative outcomes, especially among the Slovenian AYCs. This suggests that, in line with the literature, the affection and willingness to give back the care and love received during their earlier childhood might buffer the disruptive effects of taking care of an older disabled relative [22]. The high percentage (27.6%) of AYCs in the two country samples responding “I don’t know” to the question on the availability of formal support services indicates a low level of awareness of the role of carer among the youngsters themselves [61] and confirms the appropriateness of the classification by Leu and Becker [36].

Results showed that Slovenian AYCs receiving formal support reported twice the hours of care on average and more and higher negative caregiving outcomes than AYCs not receiving formal support. They could also count on informal help, suggesting that the mix of formal and informal support in Slovenia is not sufficient to fully address the support needs of AYCs and their older family members with LTC needs [62].

Likewise, Italian AYCs who received informal help had a worse perception of their quality of life. This confirms that the dearth of home health care services to older people with LTC needs is often counteracted by the informal care network [63], suggesting that the informal care network is not sufficient to improve the quality of life of AYCs caring for their GrPs.

This study confirms that in the two study countries, adolescents are part of older and disabled people’s informal care networks, and that taking on the role of carers can often follow a family code ethic according to which GrPs are expected to look after younger grandchildren and older grandchildren are expected to take care of their frail GrPs [62,63,64,65,66,67]. Moral rules and principles indeed differentiate good and less good behaviour and describe obligations that family members have for each other. In this way, moral rules lay the foundations for cooperation and co-existence among family members [68]. From this perspective, adolescents, living immersed in a familialistic caring culture, may take on the role of carers in order to identify and affiliate with others in the family, representing their first cultural group [68]. To live in such a culture since birth can prevent AYCs from identifying themselves as “young caregivers”, as they may take their involvement in caring tasks for granted and it can lead to defining caregiving as a “common” and “natural” commitment. This entails many difficulties in AYCs’ identification, involvement, and engagement in support programmes [69].

Moreover, in countries with a lack of a well-developed LTC, i.e., with poor home care service supports to older people and informal carers, often the whole family is involved in providing care to a sick or disabled older family member, even more so when the latter goes to live in their adult children’s home, where their grandchildren also live. In this case, the entire family is concerned, and caregiving commitments affect family roles and every individual family member by making changes in responsibility, personal privacy, proximity, and frequency of contacts with household relatives including GrPs [60,70]. In this framework, adolescents are often involved in caregiving tasks as part of a multigenerational caring family. In fact, youngsters and adolescents can be an indispensable resource for their parents, who endeavour to reconcile paid work with the care of children and parents at the same time, in welfare regimes full of flaws. This is the case for Italy and Slovenia, who both have familialistic welfare regimes where formal care for older people ageing in the community is poorly developed and families supporting ageing in place have to provide a vast amount of informal care, mostly intergenerational [60,61,62]. Moreover, in Slovenia, adult children have the lawful obligation to financially contribute for potential formal care of their older and dependent parents [71] and involving grandchildren in caring activities may represent an attempt to save economic resources that would otherwise be allocated to the purchase of private health care.

The qualitative findings confirmed the quantitative results and shed light on the specific difficulties encountered by Italian and Slovenian AYCs of GrPs. In fact, Italian AYCs mainly underlined communication problems and asked for material support for managing practical caring tasks that fatigued them from both a physical and relational point of view.

Conversely, Slovenian AYCs largely reported difficulties in the psychological realm and in managing GrPs’ behavioural disturbances, and such strains are mirrored by the request for emotional support. In fact, Slovenian AYCs living in multigenerational households could be exposed to the stress arising from the caregiving environment and especially from the behavioural disturbances of GrPs with mental illness, Alzheimer’s disease, or other types of dementia [72].

The study findings mirror the LTC systems operating in the two countries by underlining the dearth of actual formal support services available for AYCs, especially in Slovenia [21,22,23,24,25,26,27], further confirming the classification of Leu and Becker [36]. The study also advances this classification, highlighting the effects that the lack of support services and policy targeted at AYCs have on the latter at country level. In fact, the status of “emerging” country for Italy mainly translates into the need for material support among AYCs, while the status of “awakening” for Slovenia results mainly in the need of moral and emotional support for AYCs.

The sources of support requested by AYCs also reflect the LTC system where they live and grow up. In fact, Italian AYCs asked for monetary transfers probably because they are aware that this is the most common formal support that they can receive [25,40]. Similarly, given the lack of formal support, Slovenian AYCs requested emotional support from friends.

Furthermore, the quantitative analysis showed that some Slovenian AYCs had school attendance difficulties and qualitative findings revealed that the source of such a difficulty stands in the continuous requests for help by the care recipient and/or the family environment.

Finally, it is noteworthy that, according to qualitative findings, among the Slovenian AYCs there also emerged some gender care stereotypes, for example in the case of a grandmother refusing the help from her male grandchild and expecting help from the female grandchild involved in this study.

### 4.2. Policies and Support Interventions Targeting AYCs of GrPs in Italy and Slovenia

The study results enable the identification of key recommendations for policy and practice in the two participant countries.

Home health care services should be improved in Italy and especially in Slovenia, prioritizing older people with dementia, whose behavioural problems represent an extra source of burden for AYCs.

Slovenian AYCs’ difficulties in reconciling care activities with school attendance call for training meetings for teachers, school managers, social workers, and psychologists to sensitize them to the phenomenon of AYCs in order to recognize and offer/refer them to appropriate sources of support, e.g., distance (online) lessons, and/or by granting flexibility in getting to and from school and in doing homework. Moreover, the psychological and emotional strains of Slovenian AYCs calls for psycho-educational interventions that can foster the AYCs’ resilience such that they can better manage their emotions and mitigate risks to their mental health and well-being [73].

Italian AYCs, experiencing practical and material difficulties and wishing to care for their GrPs could benefit from responsive education and learning opportunities related to geriatric care and age-related issues, e.g., how to communicate with older people with dementia, how to move an “un-cooperative” disabled person, and what to do in case of an emergency.

Major attention should be reserved for AYCs co-habiting with their GrPs, because they are more exposed than non-cohabiting carers to higher intensities of caring and to the subsequent risk of poorer health outcomes as confirmed by quantitative and qualitative data.

Moreover, a follow-up of this MM study should be carried out for understanding to what extent the COVID-19 outbreak affected the caregiving activities of AYCs of GrPs and their mental well-being, considering that older people were those identified as being at a greater risk of mortality as a result of contracting the COVID-19 virus and that the youngsters were often the most penalized population group in terms of physical distancing [74,75,76]. Such a follow-up may also shed light on if and to what extent (if any) the outbreak changed the AYCs’ perceived difficulties and expressed needs for support.

### 4.3. Study Limitations

This study is not without limitations. We acknowledge the small sample size and the fact that AYCs were solely recruited in schools in both countries. This convenience sample makes it unfeasible to generalize from the study results. Thus, randomized controlled trials involving large samples are needed in future research to study the experience of AYCs of GrPs, also from a gender perspective.

At the same time, qualitative studies can bring an added value to the research by shedding light on and deepening the real-life experiences of this still largely neglected group of informal carers.

## 5. Conclusions

Italian AYCs of GrPs experience difficulties in performing practical caregiving tasks and in communicating with care recipients. Slovenian AYCs are exposed to psychological and emotional stress coming from co-habiting in multigenerational households, which may include GrPs with Alzheimer’s disease or other forms of dementia. Both groups of AYCs are impacted negatively by the lack of formal support existing in both countries. Moreover, the informal help they receive is not sufficient for counteracting the negative impacts of caregiving on their quality of life. Clearly, AYCs are influenced by their respective national LTC systems and this is mirrored by their expectations about their desired supports. Slovenian AYCs mainly requested emotional support from informal care network members, while Italian AYCs expected to receive material support and monetary transfer from the Government.

The study findings confirm the classification of Italy and Slovenia as “emergent” and “awakening” countries, respectively, in terms of awareness, service supports, and policy. They also enrich this classification by shedding light on the effect of the dearth of appropriate legislation and support on AYCs’ lives.

The results call for policy recognizing and supporting AYCs, and in the specific case of AYCs of GrPs, for measures strengthening the provision of home healthcare services for older people with LTC needs in both countries, though mainly in Slovenia. Moreover, it is important to note the need for training boosting adolescents’ knowledge, skills, and competences for managing and communicating with older people with disabilities, especially in Italy and for caring for older people with dementia, especially in Slovenia. Likewise, it is important to provide training opportunities for school staff to recognize AYCs and provide/refer to appropriate sources of support. Psychoeducational interventions that can improve AYCs’ resilience are welcomed in both countries.

While this article was being fine-tuned, a new country classification was published by Leu et al. [77], where Italy passed from the “emerging” to the “preliminary” position and Slovenia is still classified as an “awakening” country. The increase in awareness and policy responses in Italy are attributed to the local and international research on the topic of AYCs together with the action of NGOs at local level. In Slovenia, where such cooperation has not yet taken place, the awareness level and the policy response still remain low. This result encourages researchers to design further research studies on (A)YCs (of GrPs) in full cooperation with local, national, and international NGOs and to do so focusing on the needs of all family members, i.e., young, adult and older ones, in order to broaden the spectrum of policies and make them less sectoral and age-led and more global and intergenerational. Family care, in fact, cannot be an individual matter by definition.

## Figures and Tables

**Table 1:** Respondents’ characteristics by country and GrPs’ disease(s).

	AYCs			
	Total	Italy	Slovenia	*p*
	162 (100%)	87 (53.7%)	75 (46.3%)	
Gender, n (%)				<0.001
Male	30 (18.5%)	27 (31.0%)	3 (4.0%)	
Female	128 (79.0%)	58 (66.7%)	70 (93.3%)	
Transgender/non-binary	4 (2.5%)	2 (2.3%)	2 (2.7%)	
Age, n (%)				0.006
15	10 (6.2%)	10 (11.5%)	0 (0.0%)	
16	75 (46.3%)	35 (40.2%)	40 (53.3%)	
17	77 (47.5%)	42 (48.3%)	35 (46.7%)	
Country of birth, n (%)				0.020
National	156 (96.3%)	81 (93.1%)	75 (100.0%)	
Abroad	6 (3.7%)	6 (6.9%)	0 (0.0%)	
Living with the grandparent(s), n (%)	65 (40.1%)	17 (19.5%)	48 (64.0%)	<0.001
Hours a day spent providing care, n (%)	2.5 ± 2.6	2.7 ± 2.6	2.3 ± 2.6	0.421
GrPs’ disease(s) *				
Physical disability, n (%)	102(63.0%)	54(62.1%)	48(64.0%)	0.800
Cognitive impairment, n (%)	50(30.9%)	31(35.6%)	19(25.3%)	0.157
Mental illness, n (%)	36(22.2%)	18(20.7%)	18(24.0%)	0.613
Other, n (%)	38(23.5%)	14(16.1%)	24(32.0%)	0.017
Addiction (e.g., drugs, alcohol), n (%)	12(7.4%)	7(8.1%)	5(6.7%)	0.738

* More than one choice possible.

**Table 2:** Caregiving outcomes and support services received by AYCs of GrPs.

	AYCs of GrPs			
	Total *N* = 162	Italy *N* = 87	Slovenia *N* = 75	*p*
Health problems, n (%)	55 (33.9%)	28 (32.2%)	23 (36.0%)	0.609
MACA score, mean ± SD	11.9 ± 4.9	10.8 ± 4.2	13.4 ± 5.2	0.001
PANOC-Pos, mean ± SD	14.5 ± 4.1	13.9 ± 4.2	15.4 ± 3.7	0.048
PANOC-Neg, mean ± SD	2.8 ± 3.4	2.6 ± 3.2	3.2 ± 3.5	0.376
KIDSCREEN, MEAN ± SD	33.2 ± 6.8	34.7 ± 6.7	31.4 ± 6.5	0.004
School attendance, n (%)	15 (9.3%)	6 (6.9%)	9 (12.0%)	0.121
School performance, n (%)	13 (8.0%)	8 (9.2%)	5(6.7%)	0.290
Formal support services received by AYCs’ family, n (%)				0.003
No	98 (60.5%)	43 (49.4%)	55 (73.3%)	
Yes	33 (20.4%)	20 (23.0%)	13 (17.3%)	
I do not know	31 (19.1%)	24 (27.6%)	9 (9.4%)	
Informal help, n (%)	49 (32.2%)	28 (33.3%)	21 (30.9%)	0.748

The *p* values are from Chi-square and *t*-test as appropriate.

**Table 3:** AYCs’ condition by formal support received.

	**Formal Support Received by AYCs of GrPs**							
	**Italy**				**Slovenia**			
	**No**	**Yes**	**Don’t Know**	***p***	**No**	**Yes**	**Don’t Know**	***p***
Health problems, n (%)	13 (30.23)	9 (45)	25 (6)	0.342	17 (30.91)	9 (69.23)	1 (14.29)	0.016
Informal help received, n (%)	12 (28.57)	8 (40)	8 (36.36)	0.631	11 (20)	10 (76.92)	21 (30.88)	0.000
Hours of care provided, mean ± SD	3.05 ± 3.08	2.48 ± 1.35	2.32 ± 2.54	0.005	1.89 ± 1.64	4.73 ± 4.76	1.5 ± 0.55	0.003
Panoc-Neg, mean ± SD	2.97 ± 3.98	2.41 ± 2.62	2.00 ± 1.89	0.618	2.37 ± 2.54	5 ± 4.71	7 ± 6.56	0.014
	**Informal help received by AYCs of GrPs**							
	**Italy**				**Slovenia**			
	**No**	**Yes**	**Total**	***p***	**No**	**Yes**	**Total**	***p***
Health problems, n (%)	16 (28.57)	11 (39.29)	27 (32.14)	0.322	14 (29.79)	12 (57.14)	26 (38.24)	0.032
Formal help	12 (21.43)	8 (28.57)	20 (23.81)	0.631	44 (93.62)	11 (52.38)	13 (19.12)	0.000
KIDSCREEN	35.98 ± 7.39	32.63 ± 4.15	34.86 ± 6.66	0.032	31.11 ± 6.64	32.28 ± 6.52	31.44 ± 6.57	0.529

The *p* values are from Chi-square and *t*-test as appropriate.

**Table 4:** Categories and sub-categories related to support needs of AYCs of GrPs.

Categories and Sub-Categories	Number of Quotations	
	Italian AYCs	Slovenian AYCs
Support not required or underlined	19	6
Support not required	15	3
Don’t know	4	3
Material support	13	2
General support	5	0
Financial support	3	0
Physical support	2	0
Information and advice	2	1
Experts’ advice	1	1
Emotional and moral support needed	8	13
External, interpersonal support	4	5
Emotional support	4	8
Other supports needed	5	0
Recognition/legislation support	2	0
Educational support	2	0
Mental health support	1	0

**Table 5:** Categories and sub-categories related to difficulties encountered by AYCs of GrPs.

Categories and Sub-Categories	Number of Quotations	
	Italian AYCs	Slovenian AYCs
Material difficulties	41	15
Difficulties with moving and handling the care recipient	33	12
Lack of time	3	0
Financial difficulties	2	0
Lack of information	1	0
Difficulties in managing therapy	1	1
Difficulties in helping parent’s life–work balance	1	0
School problems	0	2
Communication difficulties	29	0
Difficulties in conversing, talking together	20	0
Difficulties in understanding care recipient’s requests	6	0
Difficulties in understanding care recipient’s problems	3	0
Emotional and psychological difficulties	12	4
Feeling of discomfort, sadness, and guilt	4	0
Fear of not managing to take care	4	0
Difficulties in supporting the care recipient’s mood	4	1
Difficulties in managing behavioural characteristics	0	3
Unspecified difficulties	0	5

## Data Availability

In accordance with the ME-WE project’s data management plan, data collected by means of open-ended “ad hoc” questions will not be shared. The decision that these data cannot be made publicly accessible is based both on legal and contractual restrictions, namely an increased possibility of identifying individual participants through their answers.
